# Validation of the Impact of Event Scale With Modifications for COVID-19 (IES-COVID19)

**DOI:** 10.3389/fpsyt.2020.00738

**Published:** 2020-07-28

**Authors:** Lauranne Vanaken, Sara Scheveneels, Eline Belmans, Dirk Hermans

**Affiliations:** Center for Learning Psychology and Experimental Psychopathology, Faculty of Psychology and Educational Sciences, KU Leuven, Leuven, Belgium

**Keywords:** COVID-19, coronavirus, impact of event scale, trauma, intrusion, avoidance, confirmatory factor analysis

## Abstract

Viral outbreaks can be experienced as disruptive and can be associated with trauma-related stress symptoms. In the current study, we adjusted the Dutch version of the Impact of Event Scale (IES) to assess traumatic stress symptoms related to the impact of the COVID-19 outbreak. The psychometric properties of this Impact of Event Scale with modifications for COVID-19 (IES-COVID19) were investigated by administering the IES-COVID19 to 380 university students who participated during the early stage of the COVID-19 outbreak, upon invitation *via* e-mail. Using confirmatory factor analysis, the factor structure of the IES-COVID19 was found to be similar to the original IES, indicating two latent factors: intrusion and avoidance, *χ^2^*(85) = 147.51, CFI = .92, TLI = .90, RMSEA = .044, SRMR = .049. Cronbach’s alpha showed acceptable internal consistency of the total IES-COVID19, *α* = .75. Pearson’s correlations of the IES-COVID19 over time were also sufficient, demonstrating adequate test–retest reliability, *r* = .62. Significant correlations (ranging between .15 and .50) between the IES-COVID19 and symptoms of depression, anxiety, stress, stress-related rumination, as well as negative social interactions, demonstrate adequate convergent validity. Overall, the IES-COVID19 shows to be a valid and reliable measure that can be utilized to investigate trauma-related stress symptoms of intrusion and avoidance related to the short- and long-term impact of the COVID-19 outbreak.

## Introduction

There is a wide consensus that during times of pandemic not only physical health, but also mental health is affected. In particular, many people exhibit depressive, stress- and anxiety-related symptoms in response to viral outbreaks and quarantine measures. For instance, during the H1N1 influenza outbreak (swine flu), 15% of a general population sample reported to feel worried about contracting H1N1, and 6% experienced emotional distress (1). Similarly, during the severe acute respiratory syndrome (SARS) epidemic in 2003, health care workers, individuals diagnosed with SARS, and people exposed to SARS patients exhibited depressive as well as anxiety- and stress-related symptoms (2, 3). Among these stress symptoms, trauma-related stress symptoms were found to be common in 10 to 36% of health care workers and diagnosed SARS patients (3–7). Notably, these trauma-related stress symptoms persisted over time and were still present 13 to 26 months after the outbreak (8). Similar findings have been reported during the Middle East Respiratory Syndrome (MERS) outbreak: 7.8% of healthcare workers who were involved in the treatment and diagnosis of MERS exhibited trauma-related stress symptoms (9).

To reduce transmission rates during viral outbreaks, physical distancing measures are taken, and people are encouraged to stay at home. Empirical evidence that focusses on the impact of quarantine measures and isolation during viral outbreaks demonstrates that these measures are in particular associated with negative psychological effects, including, depression, irritability, insomnia, confusion, and anger (3, 10). In addition, a large amount of evidence indicates that being quarantined is also associated with trauma-related stress symptoms (10). During the SARS outbreak, symptoms of trauma-related stress were observed in 28.9% of quarantined respondents (3). Moreover, being quarantined predicted trauma-related stress symptoms three years later (11). In the context of the H1N1 influenza outbreak (swine flu), one study showed that individuals who were quarantined reported trauma-related stress levels that were four times higher compared to those who were not quarantined (12).

Emerging findings on the COVID-19 outbreak suggest a similar psychological impact as in previous viral outbreaks [*e.g.*, (13, 14)]. Mertens and colleagues (2020) conducted an online study three days after the World Health Organization declared the coronavirus outbreak a pandemic (15). Respondents reported a wide range of concerns regarding the COVID-19 outbreak. Li and colleagues (2020) examined differences in negative and positive emotions before and after the declaration of the COVID-19 epidemic and found that anxiety, depression, and anger increased, while positive emotions and life satisfaction decreased (16). In addition to depressive symptoms, worrying and anxiety, evidence reveals that trauma-related stress symptoms were common during the initial stages of the COVID-19 outbreak (*e.g.*, (14)). A study from Li et al. (2020) shows that both the general public and health care staff might suffer from vicarious traumatization (17). Nonmedical health care workers reported more trauma-related stress compared to medical health care workers (18). Moreover, the presence of physical COVID-19 symptoms was found to be associated with higher trauma-related stress symptoms in health care workers (19). In a study on the psychological impact during COVID-19 experienced by psychiatric patients, Hao et al. (2020) found that more than one-third of psychiatric patients might fulfill the diagnostic criteria of posttraumatic stress disorder (PTSD) (20).

In conclusion, evidence on previous viral outbreaks as well as emerging findings on the current COVID-19 outbreak indicates that viral outbreaks and taken quarantine measures are commonly experienced as disruptive or traumatic. Trauma-related stress symptoms are an important aspect of the psychological impact of a viral outbreak. Moreover, in previous outbreaks these trauma-related stress symptoms persisted in the long run. Future research will reveal whether a similar long-term impact is found in the aftermath of the COVID-19 outbreak. Notably, Belgium, the country where this study was conducted, is severely affected by the COVID-19 outbreak. By the second half of June 2020, about 60,000 COVID-19 cases were confirmed, and 9,600 people died from COVID-19 in Belgium (21).

To measure traumatic stress symptoms in the context of viral outbreaks, the Impact of Event Scale has found to be valuable [IES; (22)]. The IES is a self-report scale assessing subjective distress related to a specific life event. The 15-item scale was developed to assess two dimensions that characterize responses to traumatic stressors: seven items to measure intrusions (intrusively experienced ideas, images, feelings, or bad dreams) and eight items to measure avoidance (self-reported avoidance of ideas, feelings or situations). For the Dutch version of the IES (23), a factor and cluster analysis confirmed the two dimensions (23, 24) and both subscales indicated high internal consistency (*α* = .93 for intrusion and *α* = .90 for avoidance; (24)). In addition, the Dutch IES shows adequate convergent validity with the highest correlations between the IES and the anxiety and depression subscales of the SCL-90 (24). An important advantage of the IES, in comparison with other self-report measures of psychological impact, is that the event can be specified. The current study aims to investigate whether the IES can be used to measure the psychological impact of the COVID-19 outbreak. We adjusted the Dutch translation of the IES and developed the Impact of Event Scale with modifications for COVID-19 (IES-COVID19). We examined the psychometric properties of the IES-COVID19 by administering it during the early stages of the COVID-19 outbreak in a sample of university students. Using confirmatory factor analysis, we tested whether a similar factor structure as in the IES emerged (24). In addition, internal consistency, test–retest reliability and convergent validity of the IES-COVID19 were evaluated.

## Methods

### Participants

At the first timepoint (T1), a total of 380 students at KU Leuven took part in the study, 335 (88.16%) women and 45 (11.84%) men, after e-mail invitation *via* the university’s Experiment Management System (EMS). Their average age was *M* = 19.44, *SD* = 1.40, *range* = 17–28. All 380 participants were invited *via* e-mail (using EMS) one month later to participate in the follow-up measurement. At Timepoint 2 (T2), 246 respondents took part (64.74% response rate), 221 (89.84%) women and 25 (10.16%) men. They averaged at an age of *M* = 19.51, *SD* = 1.31, *range* = 18–27. Data were sampled from two subgroups (A and B) of participants at each timepoint (T1: n_AT1_ = 198, n_BT1_ = 182; T2: n_AT2_ = 123, n_BT2_ = 123). Belonging to subgroup A or B was a consequence of both of the first authors having access (contact details) to students that were in different years of their education (third-year [group A] and first-year [group B] psychology academic bachelor students). The groups did not differ in terms of gender, *p* = .88; however, participants of group A were on average 1.36 years older than participants of group B, *t*(378) = 10.79, *p* <.001. Nonetheless, the two samples were considered comparable, since both consisted of predominantly young, female university (psychology) students. Procedural elements were kept similar over groups, groups only differed in in the administration of specific questionnaires to test the convergent validity of the IES. Group A filled out the DASS-21, IES-COVID19, FS, and SSL, whereas group B filled out the DASS-21, IES-COVID19, and SRRS (see abbreviations in the *Measures* section). Timing of administration was identical, as both groups filled out the questionnaires in the same weeks.

### Measures

#### The Impact of Event Scale With Modifications for COVID-19 (IES-COVID19)

The IES-COVID19 was developed based on the Dutch version of the Impact of Event Scale (Brom & Kleber, 1985). Items 1, 4, 5, 6, 10, 11, and 14 concern the Intrusion subscale. Items 2, 3, 7, 8, 9, 12, 13, and 15 are part of the Avoidance subscale. Every item is rated on a 4-point scale ranging from ‘not at all’(0) over ‘seldom’(1) and ‘sometimes’(3) to ‘often’(5). Higher scores indicate a higher psychological impact of the situation with regard to COVID-19. Subscale scores are calculated by summing the respective items and the total IES-COVID19 score is calculated by the sum of all of the 15 items. The instructions of the IES were adapted to specifically apply to ‘the situation with regard to COVID-19’. The items were largely kept similar to the original 15-item IES. Only when references to the past were made in the original version (*e.g.*, reminder, memory, still, …), we changed the item to match present times (*e.g.* thoughts, thinking), as the COVID-19 pandemic was ongoing during data collection. The items and full instructions of the IES-COVID19 are included in **Appendix 1**.

#### The Depression Anxiety and Stress Scales (DASS-21)

To investigate symptoms of internalizing psychopathology, we used the Dutch version of the Depression, Anxiety and Stress Scales [DASS-21: (25); Dutch translation: (26)]. This self-report instrument assesses symptoms of depression, anxiety and stress. Every item is to be rated on a 4-point Likert scale, ranging from ‘not at all or never applicable’ (0) to ‘definitely or very often applicable (3). Higher scores thus indicate higher rates of depression, anxiety, and stress. It has proven to be internally consistent, .85 ≤ Cronbach’s *α* ≤ .91, test–retest reliable*, *.74 ≤ *r* ≤ .85, and shows adequate validity in a Dutch sample of first-year university students (*N* = 289) which is comparable to our sample (26).

#### Psychological Well-Being (PWB)

Psychological well-being was investigated using the Flourishing Scale [FS: (27); Dutch translation: (28)]. This self-report instrument consists of eight items to measure psychosocial prosperity and has shown to be related to the longer version of the psychological well-being scales that Ryff (1989) created (29). Each item is rated on a 7-point Likert scale, ranging from ‘strongly disagree’ (1) to ‘strongly agree (7). Higher scores thus indicate higher psychological well-being. The FS is a brief measurement of psychological well-being that has proven to perform well, with high internal reliability, Cronbach’s *α* = .86, and high temporal stability, *r* = .71 (27).

#### The Social Support List (SSL)

Perceived social support was assessed using the Social Support List-Interactions (SSL-I) & -Negative Interactions (SSL-N) (30). Both the SSL-I and the SSL-N are rated on a 4-point Likert scale, ranging from ‘seldom or never’ (1) to ‘very often’. Since the SSL-I includes positively formulated items (*e.g.*, people support me, calm me, give me good advice, *etc*.), higher scores indicate more positive social interactions. However, the SSL-N includes negatively formulated items (*e.g.*, people blame me, treat me unfairly, don’t keep their promises to me, *etc*.), so higher scores indicate more negative social interactions. These scales have shown good construct validity, high internal reliability, SSL-I:.90 ≤ Cronbach’s *α* ≤.93; SSL-N:.69 ≤ Cronbach’s *α* ≤.81, and test–retest stability, SSL-I: *r = *.77; SSL-N: *r = *.56 (30). Research has indicated that negative interactions (*e.g.* giving one disapproving comments, treating one unfairly), are not at the other end of the spectrum of positive interactions. They are seen as an independent domain of interpersonal functioning and are related to psychological non-well-being (31).

#### The Stress-Reactive Rumination Scale (SRRS)

The SRRS (32, 33) is a 25-item self-report measure that was developed to assess three cognitive tendencies in response to major life stressors: (1) the tendency to focus on negative attributions and inferences; (2) the tendency to focus on hopeless cognitions; (3) the tendency to focus on active coping strategies and problem solving solutions. Answers are given on an 11-point scale, ranging from ‘never’ (0), over ‘half of the time’ (5), to ‘always’ (10). Higher scores indicate a stronger tendency to focus on (1) negative attributions, (2) hopeless cognitions and (3) problem solving. The negative attributions subscale shows adequate internal validity (Cronbach’s *α* = .89), test–retest reliability (*r = *.71) and convergent validity (correlations with depression and rumination scales). For this study, we instructed respondents to complete the SRRS with regard to the COVID-19 outbreak.

### Procedure

The study was conducted online during the COVID-19 outbreak. Testing at T1 occurred between March 23 and March 27, 2020, within two weeks after the World Health Organization declared the COVID-19 outbreak a pandemic. Testing at T2 took place between April 22 and April 29, 2020. At both timepoints physical distancing measures were in force in Belgium, which meant that citizens were required to stay at home and avoid contact with people outside of their household, and only essential journeys were allowed. Participants were contacted *via* email to take part in the study. In the informed consent, participants were informed about the aims and procedure of the study, and they were told that they could stop their participation at any time without further consequences. After agreeing to the informed consent, they could start completing the questionnaires. Respondents were instructed to do this in a quiet space with no distractions and to respond to all questions as honestly as possible. Group A filled out the DASS, IES-COVID19, FS, and SSL, whereas group B filled out the DASS, IES-COVID19 and SRRS. After participation, all participants were given contact details of the research team, professional help instances, and they were thanked for their effort and time. Participants either received course credit or an online voucher as reimbursement for their participation. The study was conducted in accordance with ethical guidelines and approved by the Social and Societal Ethics Committee of the KU Leuven (G-2018 10 1357 and G-2019 09 1744).

### Analyses

Data were analyzed using JASP (Version 0.12.2) and SPPS (version 26). First, we calculated means and standard deviations for each item of the IES-COVID19. In line with previous research (22, 24, 34, 35), endorsement scores were calculated for each item as well, defined as the percentage of responses larger than zero. Second, the hypothesized factor structure of the IES-COVID19 was tested using confirmatory factor analysis (CFA) (24), using Maximum Likelihood estimation. To compare the fit of different models, we inspected the chi-square fit index. Since the latter is very sensitive to sample size, we also included the Comparative Fit Index (CFI), the Tucker-Lewis index (TLI), the Root Mean Square Error of Approximation (RMSEA) and the Standardized Root Mean Square Residual (SRMR) (36). Values of .90 or higher for the CFI and TFI, and values lower than 0.06 for RMSEA and lower than 0.08 for SRMR were used as the cut-offs for a good fit between the hypothesized model and the collected data (36, 37). Model 1 emanated from one factor, containing all 15 items. Model 2 consisted of two correlated factors, with—in line with previous CFA on the IES (24) —items 1, 4, 5, 6, 10, 11, and 14 loading on the first factor (Intrusion) and items 2, 3, 7, 8, 9, 12, 13, and 15 loading on the second factor (Avoidance). Modification indices were used to adapt this model and explore possible better fits to the data, for instance by allowing error covariance between certain items. For the final model, we calculated the average variance extracted for each factor. All factor analyses were run on the total sample (*N* = 380). Third, Cronbach’s alphas and construct reliability were computed to test the internal consistency of the IES-COVID19 and its subscales. Fourth, test–retest reliability (between T1 and T2) was investigated using Pearson’s correlations. Finally, convergent validity of the IES-COVID19 was assessed by calculating Pearson’s correlations between the IES-COVID19 and the DASS-21, PWB, SSL and SRRS.

## Results

### Endorsement, Means, Standard Deviations

In **Table 1**, the percentage endorsement, defined as the percentage of responses on an item larger than zero is presented. Means and standard deviations are displayed for each item and for each subscale.

**Table 1:** Means and Standard Deviations per Item and per Subscale, Percentage Endorsement and Percentage Responses for each Scale Rating point per Item.

Item	*M*	*SD*	*% end*	*% 0*	*% 1*	*% 3*	*% 5*
1	2.37	1.69	86.80	13.20	31.80	35.00	20.00
2	2.34	1.75	85.50	14.50	33.20	30.30	22.10
3	2.22	1.88	75.80	24.20	25.80	26.80	23.20
4	1.11	1.74	39.50	60.50	16.60	10.30	12.60
5	1.98	1.84	74.20	25.80	32.40	21.60	20.30
6	0.78	1.55	27.90	72.10	12.10	6.60	9.20
7	1.35	1.85	46.80	53.20	17.90	13.70	15.30
8	2.24	1.87	77.40	22.60	27.40	26.60	23.40
9	1.17	1.80	41.10	58.90	17.60	8.90	14.50
10	1.16	1.75	44.20	55.80	21.60	9.20	13.40
11	1.93	1.83	72.90	27.10	31.80	21.80	19.20
12	1.32	1.88	47.70	52.60	22.60	7.10	17.60
13	2.08	1.96	70.00	30.00	25.30	20.30	24.50
14	1.89	2.03	60.80	39.20	21.10	15.50	24.20
15	1.29	1.77	48.70	51.30	22.10	12.90	13.70
Intrusion	11.22	7.15					
Avoidance	13.03	7.54					

The abbreviation % end indicates percentage endorsement, defined as the percentage of responses on an item larger than zero. The abbreviations %0, %1, %3, %5 respectively indicate the percentage of responses on an item being 0, 1, 3, or 5.

All results are calculated using the baseline sample (T1).

### Confirmatory Factor Analysis

Confirmatory factor analyses were performed using Maximum Likelihood estimation. In **Table 2**, the fit indices are presented for all models. The significance of the chi-square indices for all models can be attributed to our large sample size rather than a bad fit of these models to the data. This is evidenced by the values of the other fit indices (CFI, TLI, RMSEA, SRMR), which point in the direction of an adequate and increasingly better fit of the following models to the data.

**Table 2:** Fit indices for Confirmatory Factor Models of the IES-COVID19.

Model	*χ^2^*	*df*	*p*	*CFI*	*TLI*	*RMSEA*	*SRMR*
Model 1	244.80	90	<.001	.80	.76	.067	.061
Model 2	236.80	89	<.001	.81	.77	.066	.060
Model 2a	206.96	88	<.001	.84	.81	.060	.057
Model 2b	180.31	87	<.001	.88	.85	.053	.053
Model 2c	166.33	86	<.001	.89	.87	.050	.051
Model 2d	147.51	85	<.001	.92	.90	.044	.049

Comparative Fit Index (CFI), the Tucker–Lewis index (TLI), the Root Mean Square Error of Approximation (RMSEA) and the Standardized Root Mean Square Residual (SRMR).

Model 1: one-factor model. Model 2: two-factor model. Model 2a: Model 2 plus allowing for error covariance between items 3 and 13. Model 2b: Model 2a plus allowing for error covariance between items 4 and 6. Model 2c: Model 2b plus allowing for error covariance between items 13 and 14. Model 2d: Model 2c plus allowing for error covariance between items 10 and 3.

All results are calculated using the baseline sample (T1).

Model 1 included one general latent factor on which all 15 items loaded. Fit indices showed that the fit of Model 1 to the data was not sufficient yet. Especially the CFI and TLI indices of fit were below the cut-off score of .90 and the RMSEA index was above .06. We included two latent factors in Model 2, which corresponds to the original structure of the IES (22). Differences between chi-square statistics indicate that Model 2 fits the data significantly better than Model 1, Δ*χ^2^*(1, *N* = 380) = 8.00, *p* < .001. A two-factor solution is thus preferred above a one-factor structure for this dataset. However, the CFI, TLI, and RMSEA indices of Model 2 did not meet the criteria for an adequate fit yet.

Subsequently, Model 2 was further adapted in order to obtain a better fit to the data by implementing modifications following the indices of the highest values^1^. Those modification indices indicated that allowing error covariance between some of the items may result in a better fit of the model to the data. Allowing similar items to covary means that the variance which is not explained by the factors may covary because of the similarity between those items. In Model 2a, we allowed a covariance between items 3 and 13, since both items concerned attempts to not think about the situation concerning COVID-19. After implementing this modification, it became clear that Model 2a fits our data significantly better than Model 2, Δ*χ^2^* (1, *N* = 380) = 29.84, *p* <.001. Nonetheless, the fit of Model 2a was still insufficient (CFI and TLI <.90). Therefore, Model 2b was tested on top of Model 2a, by allowing items 4 and 6 to covary, as both items related to the impact of COVID-19 on sleeping. Comparing chi-square indices revealed that Model 2b fits the data significantly better than Model 2a did, Δ*χ^2^* (1, *N* = 380) = 26.65, *p* <.001. However, the fit indices of Model 2b still showed an inadequate fit to the data (CFI and TLI <.90). Consequently, we built on Model 2b, by including a covariance between items 13 and 14 in Model 2c. Again, the decision for allowing error covariance between these items was theoretically justified, as the items concerned intrusive thoughts and consequent coping processes with these thoughts. Model 2c showed to fit our dataset significantly better than Model 2b, Δ*χ^2^*(1, *N* = 380) = 13.98, *p* < .001, however not sufficient yet to pass our predetermined cut-off criteria (TLI <.90). Hence, we adjusted Model 2c according to the highest modification index again, which resulted in the inclusion of an inter-item covariance between item 10 and item 3 in Model 2d. Items 10 and 3 also both included thoughts and their associated coping strategies. Model 2d passed all cut-off criteria (CFI ≥ .90, TLI ≥ .90, RMSEA < .06, SRMR < .08) and fits the data significantly better than the previously tested Model 2c did, Δ*χ^2^* (1, *N* = 380) = 18.82, *p* < .001. The average variance extracted (AVE) for each of the factors in the final Model 2d surpassed the threshold of .50 (38), being .67 for Factor 1 (Intrusion) and .57 for Factor 2 (Avoidance). The factor loadings obtained in Model 2d are displayed in **Table 3**.

**Table 3:** Factor Loadings in Model 2d.

Subscale and item	Factor Loading
Intrusion	
1	0.62
4	0.82
5	0.84
6	0.59
10	0.86
11	0.80
14	1.11
Avoidance	
2	0.18
3	1.02
7	0.75
8	0.32
9	0.87
12	0.85
13	0.96
15	0.61

All results are calculated using the baseline sample (T1).

### Internal Consistency

Cronbach’s alphas were computed to examine the internal consistency of the total scale and subscales. The Cronbach’s alphas for the intrusion subscale, *α* = .67, the avoidance subscale, *α* = .59, as well as for the total IES-COVID19, *α* = .75, indicated an acceptable internal consistency (39). In addition, the construct reliability (CR) for the intrusion subscale, .93, for the avoidance subscale, .90, and for the total scale, .87, indicated also an adequate internal consistency (38).

### Test–Retest Reliability

Pearson’s correlations were calculated to investigate test–retest reliability for the total IES-COVID19 and its subscales. The test–retest reliability of the total IES-COVID19, *r* = .62, *p* <.001, the intrusion subscale, *r* = .47, *p* <.001, and the avoidance subscale, *r* = .54, *p* <.001 were moderate, indicating sufficient reliability over time (40).

### Convergent Validity

Pearson’s correlations between the total IES-COVID19, the intrusion subscale, the avoidance subscale and our concepts of interest were calculated. Results are presented in **Table 4**. The total IES-COVID19 scores as well as both subscales were significantly positively related to depression, anxiety, stress (as measured with the DASS-21), and stress-reactive rumination (as measured with the SRRS). This shows that individuals who experience a higher psychological impact by the COVID-19 outbreak exhibit other psychological symptoms as well, like depression, stress and anxiety, and they have a higher tendency to ruminate about it. Furthermore, the total IES-COVID19 score and the avoidance scale were positively associated with negative social interactions (as measured by the SSLN), indicating that people who experienced social contact in a more negative way and felt less supported by others, also experienced more psychological impact of COVID-19. However, IES-COVID19 scores did not relate to positive social interactions. Finally, scores on the IES-COVID19 and scores for psychological well-being were not related, suggesting two different constructs.

**Table 4:** Pearson’s Correlations Between the Total IES-COVID19, Subscales, and Other Scales.

Scale	IES-COVID19	INT	AVO
PWB	−.11	−.07	−.11
DEP	.27**	.19**	.29**
ANX	.31**	.26**	.28**
STR	.34**	.32**	.28**
SSLI	.14	.12	.11
SSLN	.17*	.13	.15*
SRRS	.50**	.46**	.45**

PWB, psychological well-being; DEP, depressive symptoms; ANX, anxiety symptoms; STR, stress symptoms; SSLI, social support list: positive interactions; SSLN, social support list: negative interactions; SRRS, stress-reactive rumination.

*p < .05,

**p < .001.

## Discussion

The goal of the current study was to investigate whether the Impact of Event Scale [IES; (22, 23)] is a valid measure of traumatic stress symptoms related to the outbreak of COVID-19. We adapted the IES to COVID-19 (IES-COVID19) and administered it in a sample of 380 university students during the COVID-19 outbreak in Belgium. Psychometric properties of the IES-COVID19 were investigated in terms of factor structure, internal consistency, test–retest reliability and convergent validity.

The results of the confirmatory factor analysis provide support for a two-factor structure in our data, containing the original subscales of intrusion and avoidance as described by Horowitz et al. (22) and later replicated in the Dutch version by van der Ploeg et al. (24). Minor modifications to the two-factor model were implemented according to the highest modification indices in order to ensure a better fit to the data, namely, inter-item correlations between items 3–13, 4–6, 1–14, and 3–10 were allowed. Our final model (Model 2d) fits the data significantly better than a unifactorial model or a bifactorial model without modifications and passes the predetermined cut-off criteria (CFI ≥ .90, TLI ≥ .90, RMSEA <.06, SRMR <.08) (36, 37). For completeness and consistency purposes, we utilized the subscales as well as the total IES-COVID19 scale in our further psychometric analyses.

The internal consistency of the total IES-COVID19 was adequate, demonstrating that the items cohesively measure trauma-related stress symptoms. The internal consistency of the subscales was acceptable as well (as measured by construct reliability). In addition, the test–retest reliability over a one-month period was good, rendering a similar rank order of individuals with regard to their trauma-related stress symptom severity over time. The average total scores on the IES-COVID19 were also compared between both timepoints, showing that respondents reported a higher impact of COVID-19 in March 2020, *M* = 24.84, *SD* = 13.02, compared to April 2020, *M* = 22.02, *SD* = 14.28, *t*(245) = 3.57, *p* <.001. This is line with previous research of Sloan (1988) who demonstrated that changes in reactions to traumatic events can be reliably measured using the IES (41). Wang et al. (2020) found that the IES-R is more sensitive to change during the COVID-19 outbreak as compared to the DASS-21 (14). Moreover, the magnitude of the standard deviation at both timepoints (*SD range* = 13.02–14.82) shows that there are large inter-individual differences in how the COVID-19 outbreak affects experienced traumatic stress symptoms. Accordingly, we suggest that the IES-COVID19 would be a useful instrument to assess not only broad population trends but also intra-individual fluctuations in traumatic stress symptoms over time. Since the impact of pandemic outbreaks and quarantine measures can be long-lasting (11), it is important to follow up on symptomatology over time, in particular for those individuals that were under extremely stressful circumstances during COVID-19, like health care workers or family members of people who contracted the disease. Horowitz and colleagues (22) indicated a 75% chance of developing posttraumatic stress disorder (PTSD), when scores on the IES are 27 or higher (22). They suggested that this score on the IES might represent the best cut-off for the probability of a PTSD diagnosis, with the advice of consulting a mental health professional when scores are 35 or above. Accordingly, the IES-COVID19 could be used preventively as an instrument to screen individuals at-risk for developing PTSD.

Furthermore, and in line with our expectations, total IES-COVID19 and subscale scores were significantly correlated with symptoms of depression, anxiety, stress, and stress-related rumination. The relations between the IES-COVID19 and scales that are developed to measure related psychological symptomatology support adequate convergent validity of the IES-COVID19. No relation between the IES-COVID19 and general psychological well-being, as measured with the Flourishing Scale [FS: (27); Dutch translation: (28)], was found, suggesting differences in underlying concepts between both measures. Finally, significant correlations between perceived negative social interactions and the total IES-COVID19 scores as well as the avoidance scores show that individuals who do not feel sufficiently supported by their social network experience more trauma-related stress symptoms. This points to the importance of social support as a possible protective factor for mental health in pandemic outbreaks (10).

Several other questionnaires have been developed to measure psychological reactions in the context of the COVID-19 outbreak, for instance the Fear of COVID-19 Scale [FCV-19S: (42)] and the COVID Stress Scales [CSS: (43)]. Notably, the FCV-19S and CSS focus on broader anxiety- and stress-related symptoms, whereas the focus of the IES-COVID19 is more specifically on trauma-related symptoms^2^. Moreover, an advantage of the IES-COVID19 is that it closely resembles the IES, which is a wide-spread and popular measure of the psychological impact of traumatic events and specifically viral outbreaks and of which the psychometric properties have been evaluated extensively. The FCV-19S has the advantage that it has been translated and validated in different languages [*e.g.*, (44–47)]. Similarly, it is recommended to validate the current modification of the IES to COVID-19 in other languages and countries to allow for cross-country comparisons. In addition, it would be interesting to compare the IES-COVID19 with other questionnaires on psychological reactions to COVID-19 in future research.

A possible limitation of the study might be the homogeneity of our sample, which consisted mostly of young female students. Nonetheless, total average scores on the IES-COVID19 were between *M* = 22.02 (April) and *M* = 24.84 (March), indicating a significant impact of COVID-19, even in a considerably healthy population (22). In line with Li et al. (2020), traumatization as a result from pandemic outbreaks might not only occur in health care workers and infected individuals, but also in the general population and in a vicarious way. Nevertheless, it seems important to evaluate the IES-COVID19 in other groups, such as high-risk and vulnerable populations (*e.g.*, health care workers, COVID-19 patients and relatives).

In conclusion, our results indicate that the IES-COVID19, an adaptation of the widely used IES, is a valid measure of traumatic stress symptoms (avoidance and intrusions) related to the COVID-19 outbreak. We see several possibilities for the further use of the IES-COVID19, for instance, to examine the long-term impact of COVID-19 and as a prognostic marker or screening instrument of individuals at risk of developing chronic complaints and PTSD.

## Data Availability Statement

The raw data supporting the conclusions of this article will be made available by the authors without undue reservation.

## Ethics Statement

The studies involving human participants were reviewed and approved by The Social and Societal Ethics Committee of the KU Leuven (G-2018 10 1357 and G-2019 09 1744). The patients/participants provided their written informed consent to participate in this study.

## Author Contributions

Conceptualization: SS, LV, DH. Formal analysis: EB, LV, SS. Methodology: LV, EB, SS. Supervision: DH. Writing—original draft: LV, SS. Writing—review and editing: LV, SS, EB, DH.

## Funding

This work was funded by a KU Leuven C1 project (C16/19/02) and an FWO research project (G070217N) awarded to Dirk Hermans.

## Conflict of Interest

The authors declare that the research was conducted in the absence of any commercial or financial relationships that could be construed as a potential conflict of interest.

## Footnotes

1 Notably, the modification indices suggested to include item 14 in both factors. However, we decided to refrain from this modification because the loading of item 14 changed to negative on the factor it was initially predicted to load on positively (Factor 1—Intrusion) after including the item in Factor 2 (Avoidance). This negative loading on Factor 1 is in terms of its content not compatible with the interpretation of this factor.

2 The CSS contains a subscale measuring traumatic stress.
