# Supplementary material for: Validation of the Impact of Event Scale With Modifications for COVID-19 (IES-COVID19)
Source: Front Psychiatry. 2020 Jul 28;11:738. doi: 10.3389/fpsyt.2020.00738 (PMC7399230; doi:10.3389/fpsyt.2020.00738)
Supplement: Supplementary file 1 [file DataSheet_1.doc]

**The Impact of Event Scale with modifications for COVID-19 (IES-COVID19)**

Naam: ………………………………………………………… Datum: ……………………………………………………….

**Please find below a list of statements regarding the situation related to the corona virus (COVID-19). Read each statement carefully and indicate to what extent it was applicable to you during the last seven days. If it did not occur, you can choose 0, which corresponds to ‘not at all’.**

|  | Not at all | Seldom | Sometimes | Often |
| --- | --- | --- | --- | --- |
| 1. I thought about it when I didn’t mean to. | 0 | 1 | 3 | 5 |
| 2. I avoided letting myself get upset when I thought about  it or was reminded of it. | 0 | 1 | 3 | 5 |
| 3. I tried to remove it from my thoughts. | 0 | 1 | 3 | 5 |
| 4. I had trouble falling asleep or staying asleep because of  pictures and thoughts about it that came into my mind. | 0 | 1 | 3 | 5 |
| 5. I had waves of strong feelings about is. | 0 | 1 | 3 | 5 |
| 6. I had dreams about it. | 0 | 1 | 3 | 5 |
| 7. I stayed away from things that made me think about it. | 0 | 1 | 3 | 5 |
| 8. I felt as if it hadn’t happened or wasn’t real. | 0 | 1 | 3 | 5 |
| 9. I tried not to talk about it. | 0 | 1 | 3 | 5 |
| 10. Pictures about it popped into my mind. | 0 | 1 | 3 | 5 |
| 11. Other things kept making me think about it. | 0 | 1 | 3 | 5 |
| 12. I was aware that I had a lot of feelings about it, but I  didn’t deal with them. | 0 | 1 | 3 | 5 |
| 13. I tried not to think about it. | 0 | 1 | 3 | 5 |
| 14. Every thought about it brought back the feelings about it. | 0 | 1 | 3 | 5 |
| 15. My feelings about it were kind of numb | 0 | 1 | 3 | 5 |

Highlighted in yellow: changes compared to the original IES (Horowitz et al., 1979).
